# Different activity patterns control various stages of Reelin synthesis in the developing neocortex

**DOI:** 10.1093/cercor/bhad210

**Published:** 2023-06-07

**Authors:** Kira Engeroff, Davide Warm, Stefan Bittner, Oriane Blanquie

**Affiliations:** Department of Neurology, Focus Program Translational Neuroscience (FTN) and Immunotherapy (FZI), Rhine Main Neuroscience Network (rmn2), University Medical Center of the Johannes Gutenberg University Mainz, 55131 Mainz, Germany; Institute of Physiology, University Medical Center of the Johannes Gutenberg University Mainz, 55128 Mainz, Germany; Institute of Physiology, University Medical Center of the Johannes Gutenberg University Mainz, 55128 Mainz, Germany; Department of Neurology, Focus Program Translational Neuroscience (FTN) and Immunotherapy (FZI), Rhine Main Neuroscience Network (rmn2), University Medical Center of the Johannes Gutenberg University Mainz, 55131 Mainz, Germany; Institute of Physiology, University Medical Center of the Johannes Gutenberg University Mainz, 55128 Mainz, Germany; Department of Dermatology, University Medical Center of the Johannes Gutenberg University Mainz, 55131 Mainz, Germany; European Medicines Agency, 1083HS Amsterdam, The Netherlands

**Keywords:** Reelin, neocortex, development, neuronal activity, neurotrophic signaling

## Abstract

Reelin is a large extracellular matrix protein abundantly expressed in the developing neocortex of mammals. During embryonic and early postnatal stages in mice, Reelin is secreted by a transient neuronal population, the Cajal–Retzius neurons (CRs), and is mostly known to insure the inside-out migration of neurons and the formation of cortical layers. During the first 2 postnatal weeks, CRs disappear from the neocortex and a subpopulation of GABAergic neurons takes over the expression of Reelin, albeit in lesser amounts. Although Reelin expression requires a tight regulation in a time- and cell-type specific manner, the mechanisms regulating the expression and secretion of this protein are poorly understood. In this study, we establish a cell-type specific profile of Reelin expression in the marginal zone of mice neocortex during the first 3 postnatal weeks. We then investigate whether electrical activity plays a role in the regulation of Reelin synthesis and/or secretion by cortical neurons during the early postnatal period. We show that increased electrical activity promotes the transcription of *reelin* via the brain-derived neurotrophic factor/TrkB pathway, but does not affect its translation or secretion. We further demonstrate that silencing the neuronal network promotes the translation of Reelin without affecting the transcription or secretion. We conclude that different patterns of activity control various stages of Reelin synthesis, whereas its secretion seems to be constitutive.

## Introduction

Reelin is a large secreted protein highly expressed by Cajal–Retzius neurons (CRs) and GABAergic neurons in the developing neocortex. Reelin fulfills a variety of developmental processes, including regulation of neuronal migration and formation of cortical layers in an inside–out manner (Caviness and Rakic 1978; D’Arcangelo et al. 1995; Ogawa et al. 1995), neurite outgrowth and branching (Del Río et al. 1997; Niu et al. 2004, 2008; Borrell et al. 2007), and maturation of neurotransmitter receptors (Groc et al. 2007; Hamad et al. 2021), before being downregulated (Schiffmann et al. 1997; Ringstedt et al. 1998). In line with this variety of developmental functions, dysregulation of Reelin levels is associated with multiple neurodevelopmental diseases. Mice heterozygous for the *reelin* gene (Rakic and Caviness 1995) are used as a model for schizophrenia (Ogawa et al. 1995; Tueting et al. 1999; Tsuneura et al. 2021) and in humans, patients suffering from autistic spectrum disorder or schizophrenia present decreased levels of *reelin* mRNA (Impagnatiello et al. 1998; Fatemi et al. 2005; Eastwood and Harrison 2006; Kushima et al. 2017). Inversely, experimental data from rodents and humans indicate that an aberrant survival of CRs and thus a failure to downregulate the overall level of Reelin is associated with epilepsy (Eriksson et al. 2001; Blümcke et al. 2002; Savell et al. 2019; Riva et al. 2023).

The Reelin protein presents 2 cleavage sites, generating 6 predicted proteins (Lambert de Rouvroit et al. 1999; Ignatova et al. 2004). While many studies have focused on the role of proteolytic cleavage on Reelin protein activity (Trotter et al. 2014; Ogino et al. 2017; Okugawa et al. 2020), little is known about the mechanisms regulating its expression. To our knowledge, only one study has investigated whether Reelin is expressed in a constitutive manner, or whether environmental factors control its rate of expression in the developing neocortex. This early study demonstrates that brain-derived neurotrophic factor (BDNF) promotes the downregulation of Reelin expression in embryonic CRs (Ringstedt et al. 1998). More recently, our lab and others have revealed the role of electrical activity in the cell death of CRs (Blanquie et al. 2016; Riva et al. 2019), providing an additional mechanism leading to the overall developmental downregulation of Reelin.

Here, we investigated whether neuronal activity regulates Reelin synthesis and secretion in developing neocortical neurons. In the first part of the work, we quantified the density of Reelin-expressing neuronal subtypes in the marginal zone of the neocortex during the first 3 weeks in vivo. In accordance with previous reports (Ogawa et al. 1995; Alcántara et al. 1998; Chowdhury et al. 2010; Ma et al. 2014; Ledonne et al. 2016), we show that CRs represent the main source of Reelin at birth and that between postnatal day (P) 0 and P5, the density of Reelin-positive neurons drops and the proportion of GABAergic neurons expressing Reelin rises 3-fold. In the second part of the work, we used neuronal cultures to investigate the role of electrical activity on the regulation of Reelin synthesis. We show that the GABA_A_ receptor antagonist Gabazine (Gbz) increases neuronal activity and promotes *reelin* transcription via the BDNF/TrkB pathway, but that this effect does not translate at the protein level. Inversely, silencing the neuronal network does not affect *reelin* transcription but promotes Reelin protein translation. In addition, for both activity patterns, we observed that the secretion rate of Reelin follows the rate of synthesis. These results demonstrate that different activity patterns have the ability to affect Reelin expression at specific stages of its synthesis, whereas the secretion of Reelin appears to be constitutive.

## Material and methods

### Animals

All experiments were conducted in accordance with National and European (2010/63/EU) laws for the use of animals in research. Neonatal C57BL/6NRj mice, born and housed in the local animal facility, were used for neuronal cultures. For quantification of Reelin-positive cells in slices, GAD67-GFP knock-in mice were used. These mice are heterozygous for the expression of GFP in cells positive for GAD67 (Tamamaki et al. 2003).

### Cortical coronal sections

Mice older than P10 were perfused transcardially with ice-cold PBS 0.01 M followed by 4% paraformaldehyde (PFA). P0 to P20 GAD67-GFP mice were decapitated, brains were removed from the skull and quickly submersed into 4% formaldehyde (ROTI®Histofix, Carl Roth) at 4°C overnight. Brains were then cryoprotected in 30% sucrose, 50 μm coronal sections were performed on a microtome (CM1325 & Frigomobil, Leica, Wetzlar, Germany) and stored at 4°C in PBS 0.01 M.

### Immunostaining

Slices were blocked 2 h at room temperature in PBS 0.01 M/7% (v/v) normal donkey serum (017-000-121, Jackson Immuno Research)/0.3% (v/v) triton (Triton X-100, Sigma-Aldrich) and incubated overnight at 4°C with a goat Reelin antibody (AF3820, R&D Systems, 1:500) diluted in PBS 0.01 M/2% bovine serum albumin (001-000-161, Jackson Immunoresearch)/0.05% sodium azide (S002, Sigma-Aldrich)/0.1% Triton. The next day, slices were washed 3 times with PBS 0.01 M, incubated for 2 h at room temperature with 4′,6-diamidino-2-phenylindole (DAPI) and Alexa Fluor 647-coupled secondary antibody (Dianova, Hamburg, Germany) diluted in PBS 0.01 M/2% bovine serum albumin/0.05% azide, washed 3 times with PBS 0.01 M and mounted in fluoromount (Sigma-Aldrich).

### Dissociated cortical cell cultures

C57BL/6 mice were decapitated at postnatal day (P) 0, brains were removed from the skull and quickly transferred into ice-cold Ca^2+^- and Mg^2+^-free HBSS (Gibco, Thermo Fisher Scientific) supplemented with 50 units/mL penicillin/streptomycin (Gibco), 11 mg/mL sodium pyruvate (Sigma-Aldrich), 0.1% glucose (Sigma-Aldrich) and 10 mM HEPES (Sigma-Aldrich). The hindbrain was removed, the 2 cerebral hemispheres were separated by a sagittal cut and the neocortex was isolated from subcortical structures. Cortices were then washed 3 times with HBSS and immersed in 0.05% trypsin/EDTA at 37°C. After 20 minutes (min), 200 U/mL of DNase (Sigma-Aldrich) were added to the solution. After 5 min, cells were rinsed with a plating medium composed of Minimal Essential Medium (Gibco) supplemented with 10% horse serum and 0.6% glucose. Mechanical dissociation of neocortical cells was performed by trituration through fire-polished pipettes. Once the tissue was dissociated, the solution was filtered using a cell strainer (Greiner Bio-One), living cells were counted using trypan blue staining (Sigma-Aldrich) and neurons were plated on coverslips or on 12-well plates (Greiner CELLSTAR) coated with poly-ornithine at an initial density of 1,500 cells/mm^2^, or on microelectrode arrays (MEA) coated with polyethyleneimine (0.05% in borate-buffered solution, Sigma-Aldrich) at an initial density of 4,500 cells/mm^2^. After 30 min, the plating medium was replaced by a medium containing Neurobasal medium A (Gibco) supplemented with 2% B27 (Gibco) and 1 mM L-glutamine (Gibco). Cells were incubated at 37°C in 95% air and 5% CO_2_ for 7 days in vitro (DIV).

### Pharmacology

At DIV 2–3, cultures were treated with 5 μM Ara-C. At DIV 7, 1 to 10 μL of drugs were added to cell cultures. For western blot experiments, medium was exchanged with phenol red-free medium the day of treatment. The following final concentrations were applied: 1 μM Tetrodotoxin (TTX) citrate (Tocris, Cat No: 1069); 10 μM gabazine (Tocris, Cat No: 1262), 10 μM 7,8-Dihydroxyflavone (DHF, Tocris, Cat No: 3826), 0.3 μg/mL recombinant human TrkB-Fc (R&D Systems, Cat No: 688-TK-100), 1 μg/mL recombinant Reelin (R&D Systems, Cat No 3820-MR-025). For control conditions, the same volume of solvent was applied.

### Fluorescent in situ hybridization

Fluorescent in situ hybridization (FISH) was performed using the ViewRNA ISH Cell Assay Kit (Thermo Fisher Scientific, Cat No: QVC0001) with probes targeting the coding sequences of *c-fos* (VB1-11874-VC), *reelin* (VB6-3198420-VC), and *gad1* (VB4–19846-VC). The manufacturer’s protocol was applied with the following modifications: cells were fixed in formaldehyde 4% (ROTIHistofix, Carl Roth) for 10 min, dehydrated in 50%, 70%, and 100% EtOH for 15 min, and stored in 100% EtOH at 4°C. The protease solution was used at a concentration of 1:6,000 and probes were used at a concentration of 1:800.

### Western blot

Cells were lysed in lysis buffer (Roche Applied Science, Complete), 1/4 volume of 4x lithium dodecyl sulfate (LDS) buffer (Thermo Fisher Scientific, Cat No: NP0007) and 1/10 volume of sample reducing agent 10x (50 mM DTT) were added and samples were boiled at 95°C for 3 min. Culture medium was concentrated using a 30-kDa cut-off filter (Millipore, Amicon Ultra-2 Centrifugal Filter Unit, Cat No: UFC203024), 1/100 volume of protease inhibitor (Thermofisher Scientific, Halt Protease-Inhibitor-Coktail x100, Cat No: 78440) was added and a Bicinchoninic acid (BCA) test was performed before adding 4x LDS buffer and sample reducing agent. Total protein concentrations were equalized using 1x LDS. Medium samples were boiled at 95°C for 3 min. Protein samples and molecular mass markers (Biorad, Cat No: 161-0394) were separated by Sodium dodecyl-sulfate polyacrylamide gel electrophoresis (SDS-PAGE) on 3–8% tris-acetate gels (Invitrogen) at 150 V for 1.5 h (cell lysate) or 90 V for 2 h (culture medium). Proteins were transferred to PVDF membranes (Roth) and electro-transferred to 0.45 μm nitrocellulose membranes (Amersham, GE Healthcare, Germany) for 1.5 h. After 1 h blocking at room temperature with 4% (w/v) milk in Tris-buffered saline (50 mM Tris, 150 mM NaCl, pH 7.6) containing 0.1% (v/v) Tween 20 (TBS-T), membranes were incubated overnight at 4°C in mouse anti-Reelin G10 (MAB5364, Millipore 1:1,000) or goat anti-GAPDH (AF5718, R&D System, 1:5,000). After 3 10-min washes in TBS-T, membranes were incubated with an horseradish peroxidase (HRP)-conjugated secondary antibodies (Jackson Immunoresearch; 1:10,000) diluted in TBS-T with 4% milk for 1 h. Enhanced chemiluminescence (ECL) detection was performed in a ChemiDoc XRS+ system and ImageLab software (Biorad). The densities of protein bands were quantified in ImageJ. The bands of the cellular fraction were normalized to the ones of GAPDH while the bands of the culture medium were normalized to a pre-stained marker (Thermo Fisher Scientific, Cat No: 26612) added to the samples.

### RNA extraction and qPCR

RNA was isolated using the RNeasy Mini Kit (Qiagen). mRNA was reverse-transcribed using the Transcriptor High Fidelity cDNA Synthesis Kit (Roche Applied Science). qRT-PCR was performed using the iTaq Universal SYBR Green Supermix (Bio-Rad) in a StepOne Plus Real-Time PCR System (Thermo Fisher Scientific). Primer sequences (all in 5′–3′ orientation) of target genes and probes are as follows: *GAPDH* (ATGCCAGTGAGCTTCCCGTTCAG and CATCACTGCCACCCAGAAGACTG); *reelin* (CCAGTCTCATGAAGAACTGCAC and GCTTGCGCATGCTAGTAACAC), *bdnf* (TGCAGGGGCATAGACAAAAGG and CTTATGAATCGCCAGCCAATTCTC). The qRT-PCR crossing points were used for relative quantification based on the ΔΔCt method and *GAPDH* was used as a reference gene.

### Microelectrode array recordings

Extracellular electrical recordings from cortical neurons were performed on microelectrode arrays (MEAs) containing 120 planar titanium nitrite electrodes with 4 internal references (120tMEA100/30iR-ITO-gr, Multi Channel Systems, Harvard Bioscience). MEAs had an electrode diameter of 30 μm and an interelectrode spacing of 100 μm. Signals from 120 recording electrodes were recorded with MC_Rack 4.6 software in a MEA 2100 system (Multi Channel Systems) at a sampling rate of 50 kHz and high-pass filtered at 200 Hz. Spikes were detected using a threshold-based detector set to a threshold of 7× the SD of the noise level (MC_Rack, Multi Channel Systems). Electrophysiological recordings were performed for 10 min in culture medium maintained at 37°C with a temperature controller (TC02, Multi Channel Systems) and in a humidified atmosphere with 5% CO_2_ controlled with a gas mixer (CO_2_-Controller 2000, PeCon). Spike datasets before and after pharmacological treatment were merged with MC_DataTool (Multi Channel Systems) and sorted with Offline Sorter (Plexon Inc., Dallas, Texas, United States) using K-means scan (KMS) method with a unit range of 1–4, followed by manual curation. Sorted electrophysiological units were then imported into Matlab 9.8 (The MathWorks Inc., Natick, Massachusetts, United States) for analysis using a custom written routine. Only units with at least 1 spike/min were considered as active units. The network firing rate was calculated as the sum of all neuron firing rates (i.e. the number of spikes recorded across all electrodes per second). Representative filtered traces were converted to HDF5 format with Multi Channel DataManager (Multi Channel Systems) and displayed in Matlab 9.8 (The MathWorks Inc.).

### Fluorescence microscopy and analysis

**Fig. 1:**
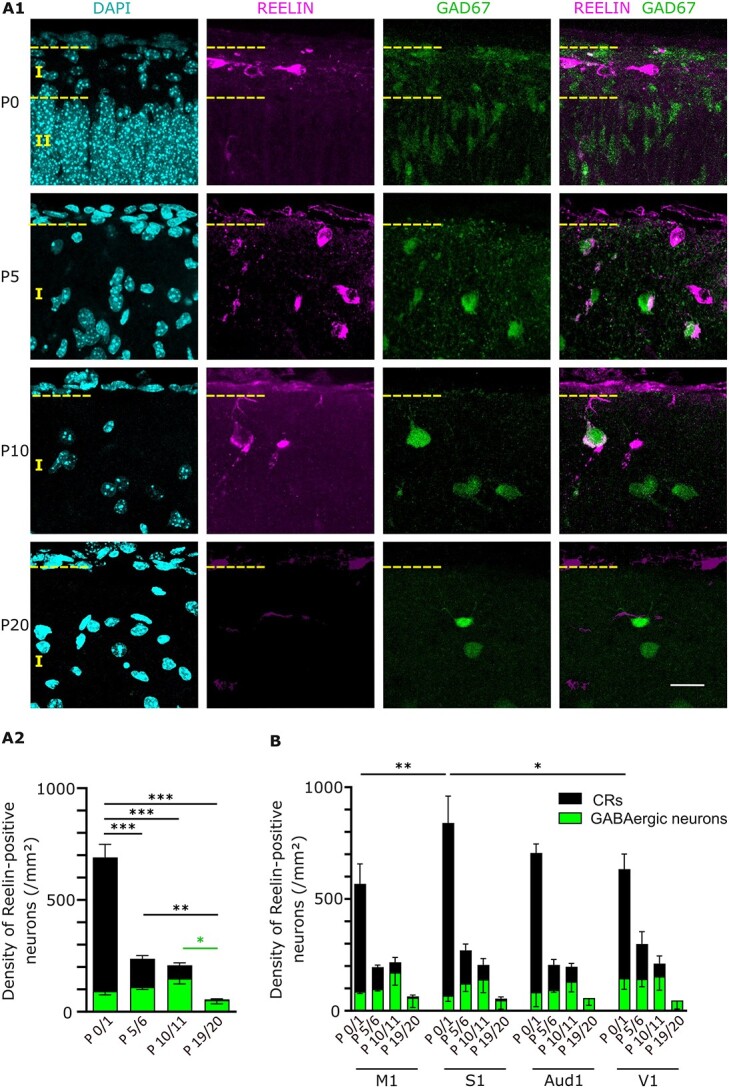
Developmental profile of Reelin expression in the marginal zone of the postnatal neocortex in vivo. (A1) Coronal sections from P0 to P20 GAD67-GFP mice were stained against Reelin, allowing the quantification of Reelin-positive GABAergic neurons (labeled in green and magenta) and CRs (labeled in magenta only). Representative images were taken with a TCS SP5 confocal microscope (Leica) using a 40x objective. A maximum projection was performed on 11 z-stacks acquired with a z-resolution of 1 μm. Scale bar is 20 μm. (A2) Quantification of the number of Reelin-positive cells in the marginal zone shows that the density of cells expressing Reelin drops during the postnatal period. At birth, CRs represent the major source of Reelin. At P5/6, the density of CRs has dramatically decreased and the proportion of GABAergic neurons expressing Reelin reaches almost half of the Reelin-expressing cells. This proportion keeps increasing until P19/20, where GABAergic neurons are the only neuronal type positive for Reelin. (B) Although the number of CRs is higher in S1 compared to M1 and V1, the dynamics of Reelin expression follows the same trend throughout the cortex, and the proportion of CRs to GABAergic neurons is similar during the first 3 postnatal weeks in all 4 cortical areas. The marginal zone/layer I is located between the 2 yellow, dotted lines. M1: primary motor cortex, S1: primary somatosensory cortex, Aud1: primary auditory cortex, and V1: primary visual cortex.

Immunostaining pictures of slices were taken with an IX81 epifluorescent microscope (Olympus) using a 20x objective. For image analysis, 2 hemispheres per animal with 4 fields of view each were analyzed. Based on DAPI staining, one field of view (FOV) was chosen per hemisphere and per cortical area, i.e. primary motor cortex (M1), primary somatosensory cortex (S1), primary auditory cortex (Au1), and primary visual cortex (V1) (Franklin et al. 2008; Paxinos et al. 2020). The marginal zone was identified based on the DAPI staining as the low-density layer located between the pial surface and the layers II–IV (see Fig. 1A1). Measurement of the marginal zone area and cell quantification were performed manually using ImageJ software. All pictures were analyzed in blind conditions.

FISH pictures were taken with a TCS SP5 confocal microscope (Leica) using a 40x objective. Z-stacks with 5 frames and a z-size of 0.75 μm. For cFOS analysis, 3 FOVs per culture and per condition were taken based on DAPI staining. For the quantification of neuronal subtypes, 6 FOVs per culture were taken. Images were analyzed with a custom-written routine in ImageJ. All pictures were taken and analyzed in blind conditions.

### Statistical analysis

All statistical tests were performed using GraphPad Prism 9.3. Normality of sample distributions was tested with Shapiro–Wilk test. When the samples were normally distributed, parametric 2-tailed paired or unpaired Student *t*-test was applied for comparison of 2 experimental groups; if more than 2 groups were compared, 1-way or 2-way Analysis of Variance (ANOVA) was performed and differences between groups were analyzed by a Tukey *post-hoc* test. If the data were not normally distributed, Wilcoxon test was performed for paired direct comparison. For MEA recording analysis, outlier testing was performed to remove units with extreme values of firing rate; rout method (*Q* = 5%) was applied after log-transforming the sample distributions. Normally-distributed data are presented as mean ± standard error of the mean (SEM) and non-normally distributed data as median with interquartile range (IQR). Data significance was considered at *P*-values < 0.05.

## Results

The density of neurons expressing Reelin declines in the marginal zone of the neocortex during the first 3 postnatal weeks and the source of Reelin switches from CRs to GABAergic neurons.

We first quantified the density of Reelin-secreting neurons in the marginal zone of P0 to P20 GAD67-GFP mice. We observed that the density of Reelin-positive cells declines from birth (Fig. 1A, P0/1: 690.5 ± 54.7 Reelin-positive cells/mm^2^, *n* = 3 animals) to P19/20 (55.6 ± 18.4 Reelin-positive cells/mm^2^, one-way ANOVA, *P* < 0.005, *n* = 3 animals), with a particularly sharp reduction in the density of Reelin-positive cells occurring between P0/1 and P5/6 (237 ± 22 Reelin-positive cells/mm^2^, 1-way ANOVA, *P* < 0.005). It should be noted that between P0/1 and P5/6, this drop is mostly due to an enlargement of the marginal zone (P0/1: 0.010 ± 0.001 mm^2^/FOV; P5/6: 0.033 ± 0.002 mm^2^/FOV, *n* = 3 animals/age group).

We next distinguished Reelin-positive GABAergic neurons from CRs based on the presence or absence of GAD67-GFP fluorescence, respectively (Causeret et al. 2021). We observed that CRs represent most of the Reelin-positive cells at birth (85.9 ± 2.7% at P0/1, *n* = 3 animals), whereas almost all reelin-positive cells are GABAergic neurons after 3 weeks postnatal (97.2 ± 1.9% at P19/20, *n* = 3 animals), with a clear switch occurring from birth (14.1 ± 2.7% of GABAergic neurons at P0/1, *n* = 3 animals) to P5/6 (46.9 ± 2.0% of GABAergic neurons, *n* = 3 animals).

We further investigated whether the decrease in the number of Reelin-positive cells varies across functional cortical areas (Fig. 1B and Supplementary Fig. S1). Although the density of CRs is higher in the S1 (772.0 ± 119.8 CRs/mm^2^ in S1) than in the other cortical areas at birth (M1: 481.7 ± 89.5 CRs/mm^2^, 2-way ANOVA, *P* < 0.005; V1: 488.5 ± 67.5 CRs/mm^2^, *P* < 0.01), the drop in the density of Reelin-positive cells occurs concurrently across all cortical areas investigated, and the proportion CRs to GABAergic neurons remains similar. These results indicate that one or several common factor(s) similarly regulate(s) the decrease in Reelin expression across the neocortex.

### Gbz application increases the level of activity in immature cortical cultures and promotes *reelin* transcription

To investigate whether electrical activity regulates *reelin* mRNA expression at a single cell level, we used primary cortical cultures and pharmacologically modulated neuronal firing. The large majority of Reelin-positive neurons in cultures undergo cell death until DIV 9 (Blanquie et al. 2016), and firing events can be first detected with the establishment of synaptic contacts towards the end of the first week in culture (Sun et al. 2010; Verstraelen et al. 2014). Therefore, to reveal activity-dependent effects on gene transcription, experiments were performed at DIV 7. Neuronal cultures deriving from GAD67-GFP mice did not exhibit fluorescence signal at this early stage and immunostainings against GAD67 did not allow the post-hoc identification of GABAergic neurons. To quantify the relative proportion of Reelin-positive neurons, FISH was performed against *reelin* and *gad 1*. At DIV 7, 63.8 ± 7% of *reelin*-positive neurons are GABAergic and 36.2 ± 7% CRs (*n* = 5 cultures).

**Fig. 2:**
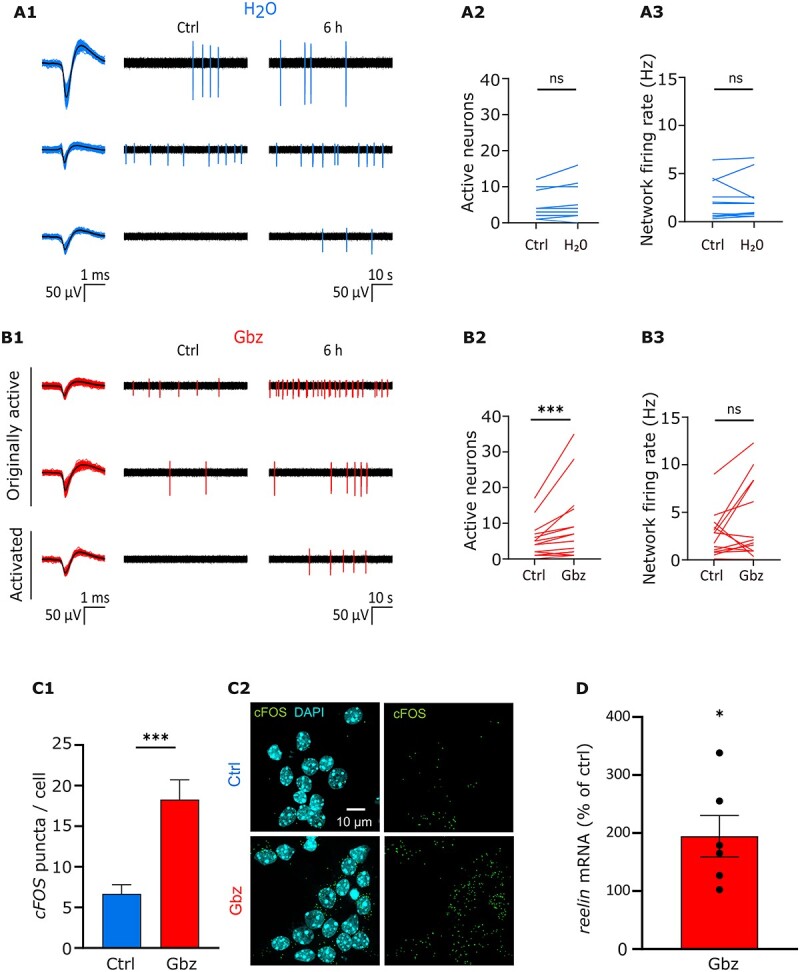
Gbz application promotes neuronal depolarization and increases *reelin* mRNA levels in immature cortical neurons. (A1) Representative electrophysiological filtered traces of independent MEA channels recorded from DIV 7 cortical cultures before (Ctrl) and after H_2_O application (6 h). Detected spikes are colored in blue and sorted spike waveforms recorded under the 2 conditions (Ctrl and 6 h) are shown superimposed (left) together with their average shape (black line). (A2) The number of active neurons remains unchanged in H_2_O-treated cultures compared to control cultures. (A3) Quantitative analysis of the network firing rate before and after treatment shows that network activity does not change upon H_2_O application. (B1) Representative electrophysiological filtered traces of independent MEA channels recorded from DIV 7 cortical cultures before (Ctrl) and after Gbz application (6 h). Detected spikes are colored in red and sorted spike waveforms recorded across both sessions (Ctrl and 6 h) are shown superimposed (left) together with their average shape (black line). (B2) Compared to control conditions, Gbz-treated cultures show an increased number of active neurons. (B3) After 6 h, Gbz treatment induces only a slight, but not significant increase in network activity rate. (C1) Quantitative analysis of FISH for *c-fos* in control versus Gbz-treated cultures. Upon Gbz treatment, the levels of *c-fos* mRNA display a 3-fold increase. The number of *c-fos* puncta is normalized to the number of DAPI nuclei. (C2) Representative pictures of a FISH performed on control cultures (upper panel) and Gbz-treated cultures (lower panel) using DAPI (blue) and *c-fos* probe (green). (D) Quantitative analysis of the levels of *reelin* mRNA in Gbz-treated cultures compared to control cultures. *Reelin* mRNA levels are normalized to the levels of *actin* mRNA. In Gbz-treated cultures, the level of *reelin* mRNA is doubled compared to untreated cultures.

**Fig. 3:**
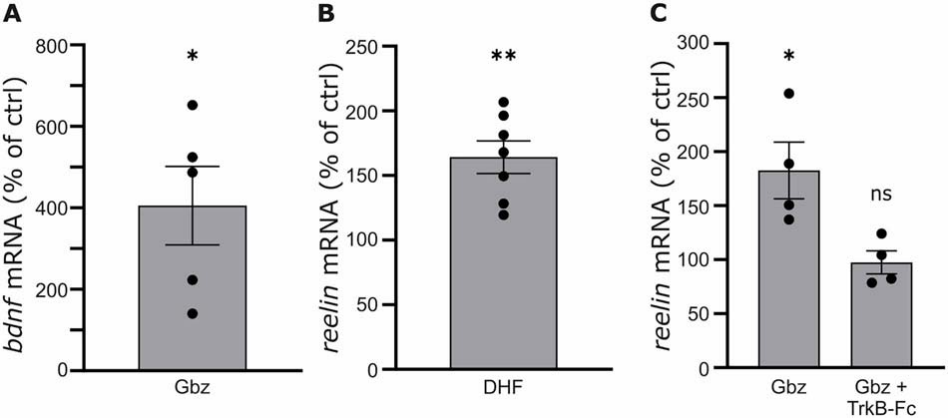
Gbz promotes *reelin* transcription via the BDNF/TrkB pathway. (A) Quantification of the relative levels of *bdnf* mRNA in Gbz-treated cultures compared to control cultures shows that Gbz promotes the transcription of the neurotrophic factor. (B) Relative levels of *reelin* mRNA in cultures treated with the agonist of TrkB receptor DHF compared to control cultures. Similar to Gbz, DHF promotes the transcription of *reelin*. (C) Relative levels of *reelin* mRNA in cultures treated with Gbz alone or Gbz + TrkB-fc compared to control cultures. Blocking the BDNF/TrkB pathway with TrkB-fc abolishes the Gbz-induced elevation in *reelin* mRNA. In all 3 experiments, mRNA levels are normalized to levels of *GAPDH* mRNA.

The effect of Gbz on the neuronal network was assessed by performing MEA recordings before and after treatment (Supplementary Fig. S2A). As a control, the same volume of vehicle (1 μL H_2_O) was applied in sister neuronal cultures. As shown in Fig. 2A and B and Supplementary Fig. 2B and C, before treatment, neuronal cultures display sparse neuronal activity with a median number of active neurons of 4 (IQR = 2–7, *n* = 27 cultures) and a median network firing rate of 1.76 Hz (IQR = 0.62–3.41). After 6 h of H_2_O application, the number of active neurons (Fig. 2A-2, H_2_O: 3.5 active neurons, IQR = 2–8.75, Wilcoxon test, *P* > 0.05, *n* = 12) and their network and single neuron firing rates (Fig. 2A-3, H_2_O: 1.44 Hz, IQR = 0.63–2.55, Wilcoxon test, *P* > 0.05; Supplementary Fig. 2B-1, Ctrl: 0.11 Hz, IQR = 0.04–0.51; H_2_O: 0.14 Hz, IQR = 0.03–0.41, Wilcoxon test, *P* > 0.05, *n* = 47) remain unchanged. Upon Gbz application, instead, the median number of active neurons increases (Fig. 2B-2, 7 active neurons, IQR = 2–14, Wilcoxon test, *P* < 0.001, *n* = 15) and the network displays only a slight but non-significant increase in the firing rate (Fig. 2B-3, Gbz: 0.24, IQR = 0.06–0.79, Wilcoxon test, *P* < 0.001, *n* = 73). Spike sorting analysis demonstrates that among the neurons originally active, application of Gbz significantly increases the firing rate (Supplementary Fig. 2C-1, Ctrl: 0.11 Hz, IQR = 0.04–0.49; Gbz: 0.24 Hz, IQR = 0.06–0.79, Wilcoxon test, *P* < 0.01, *n* = 73), but the firing frequency of activated units remains lower compared to the originally active neurons within the same dish, thereby explaining the absence of significant increase in the overall firing rate (Supplementary Fig. 2C-2, originally active neurons: 0.91 ± 0.17 Hz, activated: 0.1 ± 1.9 × 10^−2^ Hz, Student *t*-test, *P* < 0.001). Notably, the vast majority of activated neurons by Gbz (60 out of 66) is detected in previously silent channels spatially distributed across the MEA recording area. Therefore, in our model the application of Gbz not only increased the firing rate of already active neurons, but also caused a network-wide disinhibition leading to the activation of previously inactive neurons.

To confirm that the depolarizing effect of Gbz could induce changes on the transcriptional level, we performed a FISH and quantified the level of mRNA for the immediate early gene *c-fos*. Compared to control conditions (Fig. 2C, 6.7 ± 1.1 *c-fos* puncta/cell, *n* = 26 FOV from 4 cultures), Gbz-treated cultures display almost a 3-fold increase in *c-fos* mRNA (18.2 ± 2.4 *c-fos* puncta/cell, Student *t*-test, *P* < 0.005, *n* = 27 FOV from 4 cultures), confirming that Gbz application promotes neuronal activity.

We next investigated whether Gbz has any effect on the overall level of *reelin* mRNA by performing a PCR in untreated versus Gbz-treated cultures. Upon Gbz application, the level of r*eelin* mRNA is doubled compared to control conditions (Fig. 2D, 194.5 ± 35.8%, Student *t*-test, *P* < 0.05, *n* = 6 samples from 3 cultures per condition). Thus, Gbz application promotes neuronal activity and increases the transcription of *reelin* mRNA.

### Gbz-mediated increase in *reelin* transcription is mediated by the BDNF/TrkB pathway

In the rodent embryonic cortex, BDNF signaling has been shown to downregulate *reelin* mRNA transcription in CRs without affecting their cell death (Ringstedt et al. 1998). Since Gbz promotes neuronal activity in our culture model and BDNF is released in an activity-dependent manner (Brigadski and Leßmann 2020), we investigated whether and how BDNF signaling pathway could be involved in the observed Gbz-dependent increase in *reelin* transcription.

We started to assess the effect of Gbz on the levels of *bdnf* mRNA. As shown in Fig. 3A, Gbz-treated cultures present a 4-fold increase in *bdnf* mRNA levels compared to control cultures (405.3 ± 96.2%, Student *t*-test, *P* < 0.05, *n* = 4 cultures). We then tested whether the Gbz-induced elevation in *reelin* mRNA and *bdnf* mRNA are only correlated or causally-related. Neuronal cultures were treated with DHF, an agonist of the BDNF cognate receptor TrkB, and the effect on *reelin* mRNA expression was assessed. Similar to Gbz, TrkB activation leads to elevated levels of *reelin* mRNA (Fig. 3B, 164 ± 12.6%, Student *t*-test, *P* < 0.01, *n* = 4 cultures). To confirm that the Gbz-mediated increase in *reelin* expression is mediated by the BDNF/TrkB pathway, we applied Gbz while concomitantly blocking TrkB with a TrkB-Fc. As depicted in Fig. 3C, blocking the BDNF/TrkB pathway abolishes the effect of Gbz on *reelin* expression (1-way ANOVA, Gbz: 182 ± 26.1%, *P* < 0.05; Gbz + TrkB-Fc: 97.4 ± 10.6%, *P* > 0.05, *n* = 4 cultures), confirming that Gbz promotes r*eelin* mRNA expression by activating the BDNF/TrkB pathway. We conclude that increasing neuronal activity with the GABA_A_ receptor antagonist Gbz leads to an increased expression and secretion of BDNF, which then promotes *reelin* transcription upon binding on its receptor TrkB.

### Pharmacological increase of neuronal activity leads to uncoupled effects on *reelin* mRNA and Reelin protein levels

**Fig. 4:**
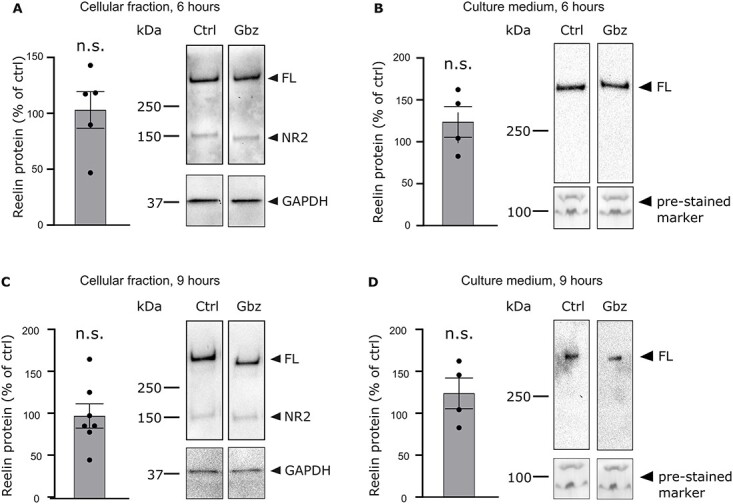
Pharmacological application of Gbz does not affect the rate of Reelin protein in the cellular fraction nor in the extracellular medium. (A) Quantification of the relative levels of Reelin protein in the cell lysate of Gbz-treated cultures compared to control cultures after a 6-h treatment period (left panel). The optical density of the Reelin band in Gbz-treated cultures is unchanged compared to control cells. The optical density of the Reelin was normalized to the optical density of the GAPDH band. The right panel shows a representative immunoblot of untreated and Gbz-treated cultures probed with anti-Reelin G10 and anti-GAPDH antibodies. (B) Quantification of the relative levels of Reelin protein in the culture medium of Gbz-treated cultures compared to control cultures after a 6-h treatment period (left panel). The optical density of the Reelin band in Gbz-treated cultures is similar to the one of untreated cultures. The optical density of the Reelin band was normalized to the optical density of a pre-stained marker. The right panel shows a representative immunoblot of the culture medium from control and Gbz-treated cultures probed with an anti-Reelin G10 antibody. (C) Same as (A) but after a 9-h treatment period. (D) Same as (B) but after a 9-h treatment period.

To investigate whether the high amounts of *reelin* mRNA transcripts found in Gbz-treated cultures are translated into proteins and secreted in the extracellular environment, western blots were performed on the cell lysate and on the culture medium of control and Gbz-treated cultures. In the cellular fraction as well as in the supernatant, cultures treated with Gbz did not present a different amount of Reelin protein compared to untreated cultures (Fig. 4A and B, cellular fraction: 103.0 ± 16.4%, Student *t*-test, *P* > 0.05, *n* = 5 cultures; culture medium: 115.2 ± 8.9%, Student *t*-test, *P* > 0.05, *n* = 4 cultures), indicating that the extra *reelin* mRNA molecules transcribed upon Gbz application are not translated into proteins. To rule out the possibility that the time window of the experiment is too small to allow *reelin* mRNA to be translated (Campo et al. 2009), we performed the same set of experiment after 9 h of pharmacological treatment. Again, after 9 h, the amount of Reelin protein is similar in Gbz-treated and untreated cultures, in the cellular fraction as well as in the medium (Fig. 4C and D, cellular fraction: 98.6 ± 14.5%, Student *t*-test, *P* > 0.05, *n* = 7 cultures; culture medium: 123.7 ± 16.1%, Student *t*-test, *P* > 0.05, *n* = 4 cultures). Thus, neuronal depolarization promotes the transcription of *reelin*, but the excess *reelin* mRNA molecules are not translated into proteins.

### Silencing the neuronal network does not affect *reelin* transcription but promotes a transient increase in Reelin translation

We next investigated whether silencing neuronal activity has the opposite effect of Gbz on Reelin expression, i.e. leading to decreased *reelin* mRNA levels without affecting Reelin protein levels. To answer this question, DIV 7 neuronal cultures were silenced with the sodium channel blocker TTX for 6 h and levels of r*eelin* mRNA and Reelin proteins were quantified.

**Fig. 5:**
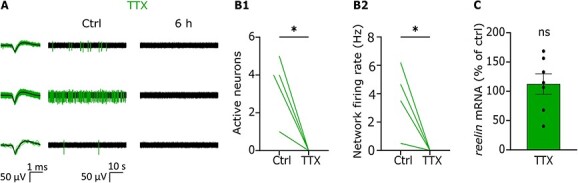
TTX application silences the neuronal network but does not affect *reelin* mRNA levels in immature cortical neurons. (A) Representative electrophysiological filtered traces of independent MEA channels recorded from DIV 7 cortical cultures before (Ctrl) and after (6 h) TTX application. Detected spikes are color labeled and sorted spike waveforms are shown superimposed (left) together with their average shape (black line). (B1-2) As can be observed by the quantification of active neurons and network firing rate, TTX fully abolishes neuronal activity after 6 h. (C) Quantitative analysis of *reelin* mRNA levels in untreated versus TTX-treated cultures shows that silencing the neuronal network does not significantly affect *reelin* mRNA levels.

To confirm the electrophysiological effect of TTX on the neuronal network, MEA recordings were performed before and after treatment. As shown in Fig. 5A and B, neuronal activity is fully abolished by TTX (Number of active neurons—Ctrl: 4, IQR = 1.75–4.75; TTX: 0, IQR = 0–0, Student *t*-test, *P* < 0.05, *n* = 4 cultures; Network Firing Rate—Ctrl: 4.09 Hz, IQR = 1.25–5.82; TTX: 0 Hz, IQR = 0–0, Student *t*-test, *P* < 0.05, *n* = 4 cultures).

We next performed a PCR and quantified the levels of *reelin* mRNA in the 2 conditions. We found a similar level of *reelin* mRNA in silenced cultures and control cultures (Fig. 5C, 112.5 ± 17.4% of control, Student *t*-test, *P* > 0.50, *n* = 4 cultures). Thus, silencing the neuronal network does not affect *reelin* transcription.

**Fig. 6:**
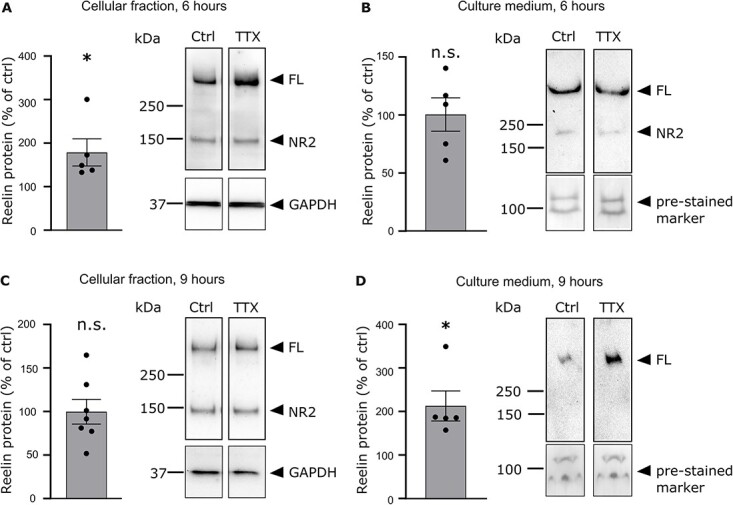
Effect of TTX on Reelin expression and secretion. (A) Quantification of the relative levels of Reelin protein in the cell lysate of TTX-treated cultures compared to control cultures after a 6-h treatment period (left panel). The optical density of the Reelin band in TTX-treated cultures is increased compared to control cultures. The optical density of the Reelin was normalized to the optical density of the GAPDH band. The right panel shows a representative immunoblot of untreated and TTX-treated cultures probed with anti-Reelin G10 and anti-GAPDH antibodies. (B) Quantification of the relative levels of Reelin protein in the culture medium of TTX-treated cultures compared to control cultures after a 6 h treatment period (left panel). The optical density of the Reelin band in TTX-treated cultures is similar to the one of untreated cultures. The optical density of the Reelin band was normalized to the optical density of a pre-stained marker. The right panel shows a representative immunoblot of the culture medium from control and TTX-treated cultures probed with an anti-Reelin G10 antibody. (C) Same as (A) but after a 9-h treatment period. (D) Same as (B) but after a 9-h treatment period.

To test whether neuronal silencing has any effect on the expression and/or secretion of the Reelin protein, neuronal cultures were treated with either TTX or H_2_O, and the amount of protein was quantified in the cellular fraction and in the supernatant. After 6 h, the mean amount of Reelin protein in the cellular fraction increases by 78.6 ± 31.3% compared to untreated cultures (Fig. 6A, Student *t*-test, *P* < 0.05, *n* = 5 cultures), whereas it remains unchanged in the supernatant (Fig. 6B, 100.5 ± 14.4%, Student *t*-test, *P* > 0.05, *n* = 5 cultures). We next performed a western blot after a 9-h treatment period. After 9 h of TTX treatment, the level of Reelin protein in the cellular fraction is back to control levels (Fig. 6C, 99.8 ± 14.2%, Student *t*-test, *P* < 0.01, *n* = 5 cultures), whereas the level of Reelin protein in the supernatant is doubled (Fig. 6D, 212.7 ± 34.5%, Student *t*-test, *P* < 0.05, *n* = 5 cultures). Thus, the extra Reelin proteins present in the cellular fraction after 6 h of pharmacological treatment are secreted in the medium after 9 h of TTX treatment, showing that the rate of secretion of Reelin strictly follows its rate of translation. Altogether, these results demonstrate that neuronal silencing does not affect r*eelin* transcription but promotes Reelin translation. In addition, upon TTX application, the secretion rate of Reelin follows its translation rate, indicating that Reelin secretion is constitutive.

## Discussion

Reelin is a secreted protein that plays major developmental roles throughout embryonic and early postnatal stages in the cortex. Here, we established a detailed pattern of Reelin expression in the developing neocortex and used dissociated cortical neurons to investigate the role of electrical activity in the regulation of Reelin protein synthesis or Reelin secretion.

We started to establish the developmental pattern of Reelin expression in the marginal zone of the neocortex. In accordance with previous reports (Schiffmann et al. 1997; Alcántara et al. 1998; Ma et al. 2014), the density of neurons secreting Reelin declines during the postnatal period and the identity of neurons synthesizing Reelin switches from mostly CRs at birth to almost exclusively GABAergic neurons after 3 weeks postnatal (Schiffmann et al. 1997; Alcántara et al. 1998; Chowdhury et al. 2010; Ma et al. 2014; Ledonne et al. 2016). Strikingly, the density of CRs drops between P0 and P5/6. These data reveal that the reduction in the density of CRs in the marginal zone occurs at the same time than the swift developmental upregulation of Reelin in deep layer GABAergic neurons (Schiffmann et al. 1997; Alcántara et al. 1998). We further found that the number of Reelin-positive neurons and the proportion of each neuronal type at each developmental stage is highly conserved across all cortical areas investigated. Altogether, these data show that the pattern of Reelin expression is tightly controlled in a time- and cell type-specific manner, and that the intrinsic and/or environmental factors controlling this developmental pattern are conserved throughout the neocortex.

During the first postnatal weeks, electrical activity influences several developmental mechanisms, including the cell death of CRs (Blanquie et al. 2016). To investigate whether the expression of Reelin is also regulated by electrical activity, pharmacological treatments were applied to dissociated cortical cultures, a model that preserves developmental features of neuronal networks and enables the control and monitoring of network activity levels (Weir et al. 2015; Warm et al. 2022). In this in vitro model, spontaneous activity can first be observed at the end of the first week (Sun et al. 2010; Verstraelen et al. 2014). At this early stage, however, neuronal cultures deriving from GAD67-GFP mice did not exhibit fluorescence signal and immunostainings against GAD67 did not allow the post-hoc identification of GABAergic neurons. Only FISH performed against *reelin* and *gad 1* allowed the quantification of the relative proportion of neuronal subtypes and showed that at DIV 7, the majority (64%) of *reelin*-positive neurons are GABAergic (*gad 1*-positive) and 36% are CRs (*gad 1*-negative).

At DIV 7, application of the GABA_A_ receptor antagonist Gbz induces neuronal depolarization throughout the network as quantified by MEA recordings and causes elevated mRNA levels of the immediate early gene *c-fos*. Interestingly, in TTX-treated cultures, where action potential generation is completely prevented, *c-fos* levels are unchanged compared to untreated cultures. Next, we measured the level of *reelin* mRNA in control, Gbz- or TTX-treated cultures. Whereas Gbz leads to a 2-fold increase in *reelin* mRNA levels, TTX does not affect the level of *reelin* mRNA transcription. Since neuronal depolarization induced by Gbz leads to elevated levels of *c-fos* and r*eelin* mRNA whereas TTX does not affect the level of either of these genes, we hypothesize that electrical activity has the ability to affect gene transcription only if a certain level of activity has been reached. We cannot exclude that in vivo the difference in activity levels between physiological conditions and a silenced network would allow a significant change in either *c-fos* or *reelin* mRNA levels.

Experiments performed in embryonic BDNF^−/−^ mice have demonstrated that the developmental upregulation of BDNF is causal for *reelin* mRNA downregulation in neocortical CRs (Ringstedt et al. 1998). Interestingly, although expression and secretion of BDNF is enhanced by electrical activity (Balkowiec and Katz 2000), inducing epileptiform activity for 24 h using either elevated potassium or kainate does not modify the overall level of *reelin* mRNA and Reelin protein in organotypic hippocampal slices (Tinnes et al. 2011). These last results are in accordance with data showing that Reelin expression is independent of activity in cerebellar neurons (Lacor et al. 2000). Here, we investigated the role of increased electrical activity and the subsequent release of BDNF on *reelin* mRNA expression in cortical neurons. We found that elevated levels of activity are associated with increased levels of *reelin* mRNA. We further found that upon Gbz application, increased *reelin* mRNA levels do not only positively correlate with *bdnf* mRNA levels, but that BDNF signaling is causal for the increase in *reelin* mRNA expression. One possibility for these discordant results is that the regulation of *reelin* mRNA expression is cell type-dependent. In our cortical cultures, about two-thirds of the Reelin-positive neurons are GABAergic and a third are CRs. The work of Ringstedt et al. focuses on the embryonic neocortex, where the large majority of Reelin-secreting cells are CRs. We hypothesize that the opposite effect of BDNF observed in embryonic CRs versus cortical cultures is due to its antithetic effect in CRs versus GABAergic neurons: BDNF might prevent *reelin* mRNA expression in CRs (Ringstedt et al. 1998) whereas it promotes *reelin* mRNA transcription in GABAergic neurons. The putative cell-type specific mechanism underlying Reelin expression is further supported in the work of Tinnes et al., where a transient increase of electrical activity does not affect Reelin protein expression in CRs, but leads to a 30% decrease in Reelin immunoreactivity in interneurons a week later.

Interestingly, silencing the neuronal network with TTX does not affect *reelin* mRNA transcription but promotes a transient increase in Reelin protein translation. Thus, increasing neuronal activity and silencing the neuronal network do not show opposite effects but rather appear to act on different steps of protein synthesis. We hypothesize that neuronal activity leads to increased *reelin* mRNA via the BDNF/TRkB pathway, thus activating transcription factors such as CREB (Shaywitz and Greenberg 1999). On the other side, neuronal silencing leads to an overall decrease in gene expression and might lead to an increased availability of translation machinery elements such as transportation elements, ribosomes, or tRNA (Metzl et al. 2020). This increased availability of translation machinery elements could allow an accelerated translation of *reelin* mRNA molecules at first (seen in the western blot as an increased protein levels) before reaching a steady-state level matching the transcriptional speed.

We further investigated whether changing activity patterns leads to a modification in the rate of Reelin secretion. After 6 h of pharmacological treatment to either silence the neuronal network or promote neuronal depolarization, no change in Reelin levels was observed in the culture medium. However, the increased levels of Reelin protein observed in the cellular fraction after 6 h of TTX application are followed by elevated Reelin levels in the culture medium 3 h later, indicating that Reelin secretion follows the level of Reelin protein translation. These data are in accordance with previous experiments performed in adult cerebellar neurons showing that Reelin secretion is not regulated by activity-dependent exocytosis (Lacor et al. 2000). On the other side, experimental data performed in a mouse model temporal lobe epilepsy demonstrate that epileptiform activity leads to an intracellular accumulation of Reelin in adult hippocampal CRs (Duveau et al. 2011). It is possible that under our conditions, the levels of activity in Gbz-treated cultures are not elevated enough to lead to a modification of Reelin protein expression and subsequent secretion.

Here, we demonstrated that the expression of Reelin is mostly ensured by CRs until the first postnatal days in vivo*,* and that in neuronal cultures, various electrical activity patterns control different stages of Reelin synthesis in developing cortical neurons. These data hint at a cell-type specific mechanism by which electrical activity switches off the synthesis of Reelin in CRs and upregulates Reelin expression in GABAergic neurons. In animal models and tissues from human patients, a defect in Reelin expression and/or secretion leads to severe neurodevelopmental disorders. It will be interesting to investigate in more details the mechanisms allowing electrical activity to modify the levels of Reelin synthesis in developing CRs and GABAergic neurons under physiological and pathological conditions. For instance, the hypermethylation of the *reelin* promoter in adult GABAergic neurons represents an important mechanism leading to the downregulation of *reelin* transcription in patients suffering from schizophrenia (Abdolmaleky et al. 2005; Grayson et al. 2006; Magwai et al. 2021). Whether electrical activity or neurotrophic factor signaling have any effect on the methylation of *reelin* promoter in developing cortical neurons would be an interesting avenue for future research (Moore et al. 2013). These findings will be important to better understand the physiological steps controlling cortical development and to gain insight on the etiology of developmental disorders associated with pathological levels of Reelin.

## Supplementary Material

Supplementary_Figure_1_bhad210

Supplementary_Figure_2_bhad210

Supplementary_Figure_4_bhad210

Supplementary_Figure_6_bhad210

## Data Availability

The datasets generated and/or analysed during the current study are available from the corresponding author on reasonable request.

## References

[ref1] Abdolmaleky HM, Cheng K, Russo A, Smith CL, Faraone SV, Wilcox M, Shafa R, Glatt SJ, Nguyen G, Ponte JF, et al. Hypermethylation of the reelin (RELN) promoter in the brain of schizophrenic patients: a preliminary report. Am J Med Genet Part B Neuropsychiatr Genet. 2005:134B:60–66. 10.1002/ajmg.b.30140 15717292

[ref2] Alcántara S, Ruiz M, D’Arcangelo G, Ezan F, de Lecea L, Curran T, Sotelo C, Soriano E. Regional and cellular patterns of reelin mRNA expression in the forebrain of the developing and adult mouse. J Neurosci. 1998:18:7779 LP–7799. 9742148 10.1523/JNEUROSCI.18-19-07779.1998 PMC6792998

[ref3] Balkowiec A, Katz DM. Activity-dependent release of endogenous brain-derived neurotrophic factor from primary sensory neurons detected by ELISA in situ. J Neurosci. 2000:20:7417 LP–7423. 11007900 10.1523/JNEUROSCI.20-19-07417.2000 PMC6772775

[ref4] Blanquie O, Liebmann L, Hübner CA, Luhmann HJ, Sinning A. NKCC1-mediated GABAergic signaling promotes postnatal cell death in neocortical Cajal–Retzius cells. Cereb Cortex. 2016:27:1644–1659. 10.1093/cercor/bhw004 26819276

[ref5] Blümcke I, Thom M, Otmar DW. Ammon’s horn sclerosis: a maldevelopmental disorder associated with temporal lobe epilepsy. Brain Pathol. 2002:12:199–211. 11958375 10.1111/j.1750-3639.2002.tb00436.x PMC8095862

[ref6] Borrell V, Pujadas L, Simó S, Durà D, Solé M, Cooper JA, Del Río JA, Soriano E. Reelin and mDab1 regulate the development of hippocampal connections. Mol Cell Neurosci. 2007:36:158–173. 17720534 10.1016/j.mcn.2007.06.006

[ref7] Brigadski T, Leßmann V. The physiology of regulated BDNF release. Cell Tissue Res. 2020:382:15–45. 32944867 10.1007/s00441-020-03253-2 PMC7529619

[ref8] Campo CG, Sinagra M, Verrier D, Manzoni OJ, Chavis P. Reelin secreted by GABAergic neurons regulates glutamate receptor homeostasis. PLoS One. 2009:4:e5505. 19430527 10.1371/journal.pone.0005505 PMC2675077

[ref9] Causeret F, Moreau MX, Pierani A, Blanquie O. The multiple facets of Cajal-Retzius neurons. Development. 2021:148(11). 10.1242/dev.199409 34047341

[ref10] Caviness VS, Rakic P. Mechanisms of cortical development: a view from mutations in mice. Annu Rev Neurosci. 1978:1:297–326. 386903 10.1146/annurev.ne.01.030178.001501

[ref11] Chowdhury TG, Jimenez JC, Bomar JM, Cruz-Martin A, Cantle JP, Portera-Cailliau C. Fate of Cajal-Retzius neurons in the postnatal mouse neocortex. Front Neuroanat. 2010:4:10. 20339484 10.3389/neuro.05.010.2010 PMC2845061

[ref12] D’Arcangelo G, Miao GG, Chen SC, Scares HD, Morgan JI, Curran T. A protein related to extracellular matrix proteins deleted in the mouse mutant reeler. Nature. 1995:374:719–723. 7715726 10.1038/374719a0

[ref13] Del Río JA, Heimrich B, Borrell V, Förster E, Drakew A, Alcántara S, Nakajima K, Miyata T, Ogawa M, Mikoshiba K, et al. A role for Cajal–Retzius cells and reelin in the development of hippocampal connections. Nature. 1997:385:70–74. 8985248 10.1038/385070a0

[ref14] Duveau V, Madhusudan A, Caleo M, Knuesel I, Fritschy J-M. Impaired reelin processing and secretion by Cajal–Retzius cells contributes to granule cell dispersion in a mouse model of temporal lobe epilepsy. Hippocampus. 2011:21:935–944. 20865728 10.1002/hipo.20793

[ref15] Eastwood SL, Harrison PJ. Cellular basis of reduced cortical Reelin expression in schizophrenia. Am J Psychiatry. 2006:163:540–542. 16513881 10.1176/appi.ajp.163.3.540

[ref16] Eriksson SH, Thom M, Heffernan J, Lin WR, Harding BN, Squier MV, Sisodiya SM. Persistent reelin-expressing Cajal–Retzius cells in polymicrogyria. Brain. 2001:124:1350–1361. 11408330 10.1093/brain/124.7.1350

[ref17] Fatemi SH, Snow AV, Stary JM, Araghi-Niknam M, Reutiman TJ, Lee S, Brooks AI, Pearce DA. Reelin signaling is impaired in autism. Biol Psychiatry. 2005:57:777–787. 15820235 10.1016/j.biopsych.2004.12.018

[ref18] Franklin M, Keith B, Paxinos G. The mouse brain in stereotaxic coordinates, compact: the coronal plates and diagrams. 3rd ed. Amsterdam, The Netherlands: Academic Press; 2008.

[ref19] Grayson DR, Chen Y, Costa E, Dong E, Guidotti A, Kundakovic M, Sharma RP. The human reelin gene: transcription factors (+), repressors (−) and the methylation switch (+/−) in schizophrenia. Pharmacol Ther. 2006:111:272–286. 16574235 10.1016/j.pharmthera.2005.01.007

[ref20] Groc L, Choquet D, Stephenson FA, Verrier D, Manzoni OJ, Chavis P. NMDA receptor surface trafficking and synaptic subunit composition are developmentally regulated by the extracellular matrix protein Reelin. J Neurosci. 2007:27:10165–10175. 17881522 10.1523/JNEUROSCI.1772-07.2007 PMC6672660

[ref21] Hamad MIK, Jbara A, Rabaya O, Petrova P, Daoud S, Melliti N, Meseke M, Lutz D, Petrasch-Parwez E, Schwitalla JC, et al. Reelin signaling modulates GABAB receptor function in the neocortex. J Neurochem. 2021:156:589–603. 32083308 10.1111/jnc.14990 PMC7442713

[ref22] Ignatova N, Sindic CJM, Goffinet AM. Characterization of the various forms of the Reelin protein in the cerebrospinal fluid of normal subjects and in neurological diseases. Neurobiol Dis. 2004:15:326–330. 15006702 10.1016/j.nbd.2003.11.008

[ref23] Impagnatiello F, Guidotti AR, Pesold C, Dwivedi Y, Caruncho H, Pisu MG, Uzunov DP, Smalheiser NR, Davis JM, Pandey GN, et al. A decrease of reelin expression as a putative vulnerability factor in schizophrenia. Proc Natl Acad Sci. 1998:95:15718 LP–15723. 9861036 10.1073/pnas.95.26.15718 PMC28110

[ref24] Kushima I, Aleksic B, Nakatochi M, Shimamura T, Shiino T, Yoshimi A, Kimura H, Takasaki Y, Wang C, Xing J, et al. High-resolution copy number variation analysis of schizophrenia in Japan. Mol Psychiatry. 2017:22:430–440. 27240532 10.1038/mp.2016.88

[ref25] Lacor PN, Grayson DR, Auta J, Sugaya I, Costa E, Guidotti A. Reelin secretion from glutamatergic neurons in culture is independent from neurotransmitter regulation. Proc Natl Acad Sci. 2000:97:3556–3561. 10725375 10.1073/pnas.050589597 PMC16278

[ref26] Lambert de Rouvroit C, de Bergeyck V, Cortvrindt C, Bar I, Eeckhout Y, Goffinet AM. Reelin, the extracellular matrix protein deficient in Reeler mutant mice, is processed by a metalloproteinase. Exp Neurol. 1999:156:214–217. 10192793 10.1006/exnr.1998.7007

[ref27] Ledonne F, Orduz D, Mercier J, Vigier L, Grove EA, Tissir F, Angulo MC, Pierani A, Coppola E. Targeted inactivation of Bax reveals a subtype-specific mechanism of Cajal-Retzius neuron death in the postnatal cerebral cortex. Cell Rep. 2016:17:3133–3141. 28009284 10.1016/j.celrep.2016.11.074

[ref28] Ma J, Yao X-HH, Fu Y, Yu Y-CC. Development of layer 1 neurons in the mouse neocortex. Cereb Cortex. 2014:24(10):2604–2618. 23680842 10.1093/cercor/bht114

[ref29] Magwai T, Shangase KB, Oginga FO, Chiliza B, Mpofana T, Xulu KR. DNA methylation and schizophrenia: current literature and future perspective. Cell. 2021:10(11). 10.3390/cells10112890 PMC8616184 34831111

[ref30] Metzl-Raz E, Kafri M, Yaakov G, Barkai N. Gene transcription as a limiting factor in protein production and cell growth. G3 (Bethesda). 2020:10(9):3229–3242. 32694199 10.1534/g3.120.401303 PMC7466996

[ref31] Moore LD, Le T, Fan G. DNA methylation and its basic function. Neuropsychopharmacology. 2013:38:23–38. 22781841 10.1038/npp.2012.112 PMC3521964

[ref32] Niu S, Renfro A, Quattrocchi CC, Sheldon M, D’Arcangelo G. Reelin promotes hippocampal dendrite development through the VLDLR/ApoER2-Dab1 pathway. Neuron. 2004:41:71–84. 14715136 10.1016/s0896-6273(03)00819-5

[ref33] Niu S, Yabut O, D’Arcangelo G. The Reelin signaling pathway promotes dendritic spine development in hippocampal neurons. J Neurosci. 2008:28:10339–10348. 18842893 10.1523/JNEUROSCI.1917-08.2008 PMC2572775

[ref34] Ogawa M, Miyata T, Nakajima K, Yagyu K, Seike M, Ikenaka K, Yamamoto H, Mikoshiba K. The reeler gene-associated antigen on Cajal-Retzius neurons is a crucial molecule for laminar organization of cortical neurons. Neuron. 1995:14:899–912. 7748558 10.1016/0896-6273(95)90329-1

[ref35] Ogino H, Hisanaga A, Kohno T, Kondo Y, Okumura K, Kamei T, Sato T, Asahara H, Tsuiji H, Fukata M, et al. Secreted metalloproteinase ADAMTS-3 inactivates Reelin. J Neurosci. 2017:37:3181 LP–3191. 28213441 10.1523/JNEUROSCI.3632-16.2017 PMC6596773

[ref36] Okugawa E, Ogino H, Shigenobu T, Yamakage Y, Tsuiji H, Oishi H, Kohno T, Hattori M. Physiological significance of proteolytic processing of Reelin revealed by cleavage-resistant Reelin knock-in mice. Sci Rep. 2020:10:4471. 32161359 10.1038/s41598-020-61380-w PMC7066138

[ref37] Paxinos G, Halliday G, Watson C, Kassem M. Atlas of the developing mouse brain. 2nd ed. Amsterdam, The Netherlands: Academic Press; 2020.

[ref38] Rakic P, Caviness VSJ. Cortical development: view from neurological mutants two decades later. Neuron. 1995:14:1101–1104. 7605626 10.1016/0896-6273(95)90258-9

[ref39] Ringstedt T, Linnarsson S, Wagner J, Arenas E, Ernfors P, Lendahl U, Kokaia Z, Ibáñez CF. BDNF regulates reelin expression and Cajal-Retzius cell development in the cerebral cortex. Neuron. 1998:21:305–315. 9728912 10.1016/s0896-6273(00)80540-1

[ref40] Riva M, Genescu I, Habermacher C, Orduz D, Ledonne F, Rijli FM, López-Bendito G, Coppola E, Garel S, Angulo MC, et al. Activity-dependent death of transient Cajal-Retzius neurons is required for functional cortical wiring. elife. 2019:8. 10.7554/eLife.50503 PMC6938399 31891351

[ref41] Riva M, Moriceau S, Morabito A, Dossi E, Sanchez-Bellot C, Azzam P, Navas-Olive A, Gal B, Dori F, Cid E, et al. Aberrant survival of hippocampal Cajal-Retzius cells leads to memory deficits, gamma rhythmopathies and susceptibility to seizures in adult mice. Nat Commun. 2023:14(1):1531. 36934089 10.1038/s41467-023-37249-7 PMC10024761

[ref42] Savell KE, Bach SV, Zipperly ME, Revanna JS, Goska NA, Tuscher JJ, Duke CG, Sultan FA, Burke JN, Williams D, et al. A neuron-optimized CRISPR/dCas9 activation system for robust and specific gene regulation. eNeuro. 2019:6: ENEURO.0495-18.2019. 10.1523/ENEURO.0495-18.2019 PMC6412672 30863790

[ref43] Schiffmann SN, Bernier B, Goffinet AM. Reelin mRNA expression during mouse brain development. Eur J Neurosci. 1997:9:1055–1071. 9182958 10.1111/j.1460-9568.1997.tb01456.x

[ref44] Shaywitz AJ, Greenberg ME. CREB: a stimulus-induced transcription factor activated by a diverse array of extracellular signals. Annu Rev Biochem. 1999:68:821–861. 10872467 10.1146/annurev.biochem.68.1.821

[ref45] Sun J-J, Kilb W, Luhmann HJ. Self-organization of repetitive spike patterns in developing neuronal networks in vitro. Eur J Neurosci. 2010:32:1289–1299. 20846326 10.1111/j.1460-9568.2010.07383.x

[ref46] Tamamaki N, Yanagawa Y, Tomioka R, Miyazaki J-I, Obata K, Kaneko T. Green fluorescent protein expression and colocalization with calretinin, parvalbumin, and somatostatin in the GAD67-GFP knock-in mouse. J Comp Neurol. 2003:467:60–79. 14574680 10.1002/cne.10905

[ref47] Tinnes S, Schäfer MKE, Flubacher A, Münzner G, Frotscher M, Haas CA. Epileptiform activity interferes with proteolytic processing of Reelin required for dentate granule cell positioning. FASEB J. 2011:25:1002–1013. 21148112 10.1096/fj.10-168294

[ref48] Trotter JH, Lussier AL, Psilos KE, Mahoney HL, Sponaugle AE, Hoe H-S, Rebeck GW, Weeber EJ. Extracellular proteolysis of reelin by tissue plasminogen activator following synaptic potentiation. Neuroscience. 2014:274:299–307. 24892761 10.1016/j.neuroscience.2014.05.046 PMC4381833

[ref49] Tsuneura Y, Sawahata M, Itoh N, Miyajima R, Mori D, Kohno T, Hattori M, Sobue A, Nagai T, Mizoguchi H, et al. Analysis of Reelin signaling and neurodevelopmental trajectory in primary cultured cortical neurons with RELN deletion identified in schizophrenia. Neurochem Int. 2021:144:104954. 33388358 10.1016/j.neuint.2020.104954

[ref50] Tueting P, Costa E, Dwivedi Y, Guidotti A, Impagnatiello F, Manev R, Pesold C. The phenotypic characteristics of heterozygous Reeler mouse. Neuroreport. 1999:10(6):1329–1334. 10363948 10.1097/00001756-199904260-00032

[ref51] Verstraelen P, Pintelon I, Nuydens R, Cornelissen F, Meert T, Timmermans J-P. Pharmacological characterization of cultivated neuronal networks: relevance to synaptogenesis and synaptic connectivity. Cell Mol Neurobiol. 2014:34:757–776. 24748115 10.1007/s10571-014-0057-6 PMC11488889

[ref52] Warm D, Bassetti D, Schroer J, Luhmann HJ, Sinning A. Spontaneous activity predicts survival of developing cortical neurons. Front Cell Dev Biol. 2022:10. 10.3389/fcell.2022.937761 PMC9399774 36035995

[ref53] Weir K, Blanquie O, Kilb W, Luhmann HJ, Sinning A. Comparison of spike parameters from optically identified GABAergic and glutamatergic neurons in sparse cortical cultures. Front Cell Neurosci. 2015:8:460. 25642167 10.3389/fncel.2014.00460 PMC4294161

