# Supplementary figures and images for: Different activity patterns control various stages of Reelin synthesis in the developing neocortex
Source: Cereb Cortex. 2023 Jun 7;33(15):9376–86. doi: 10.1093/cercor/bhad210 (PMC10393496; doi:10.1093/cercor/bhad210)

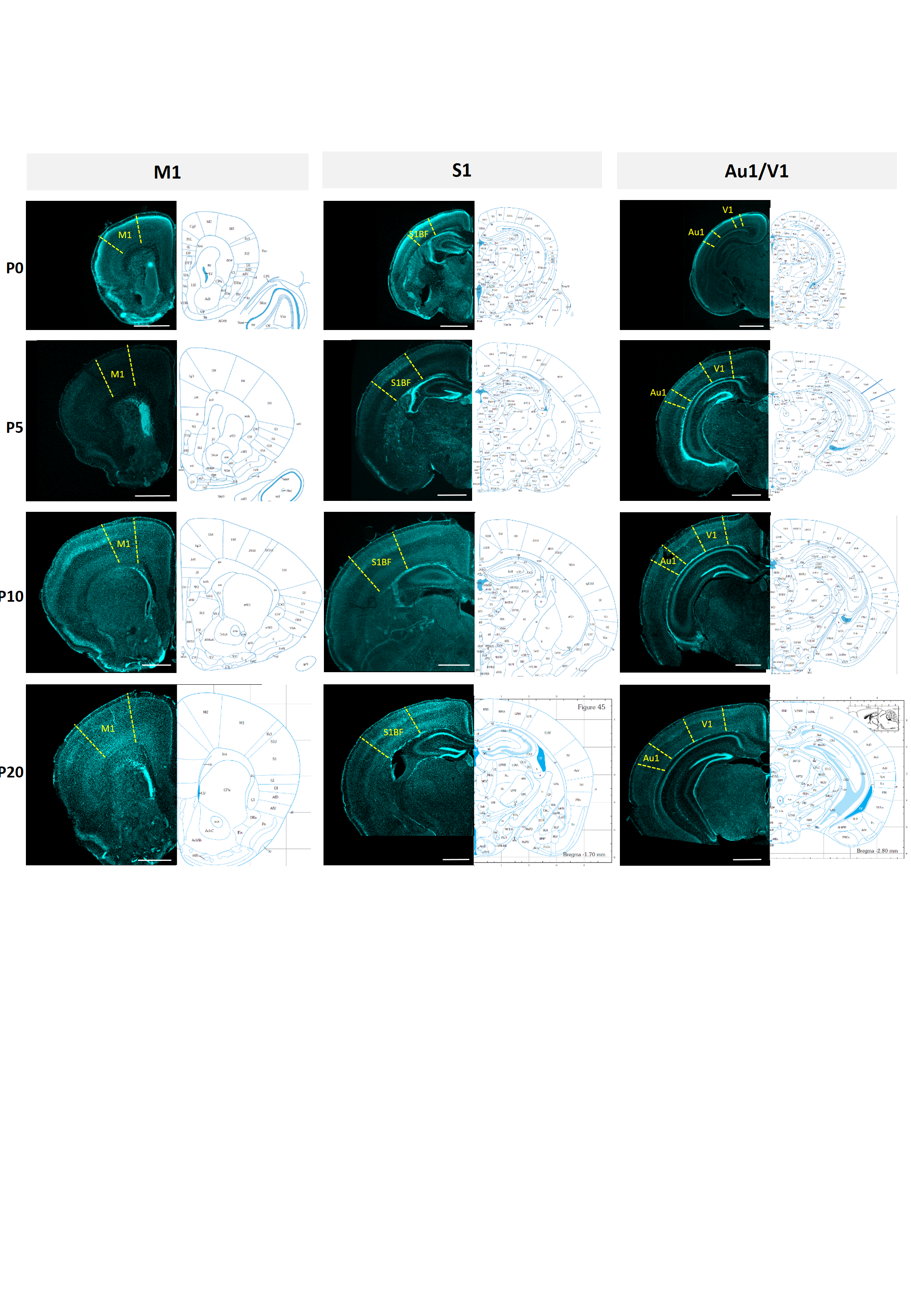

Supplement: Supplementary_Figure_1_bhad210 [file supplementary_figure_1_bhad210.zip › Supplementary_Figure_1_bhad210.tif]

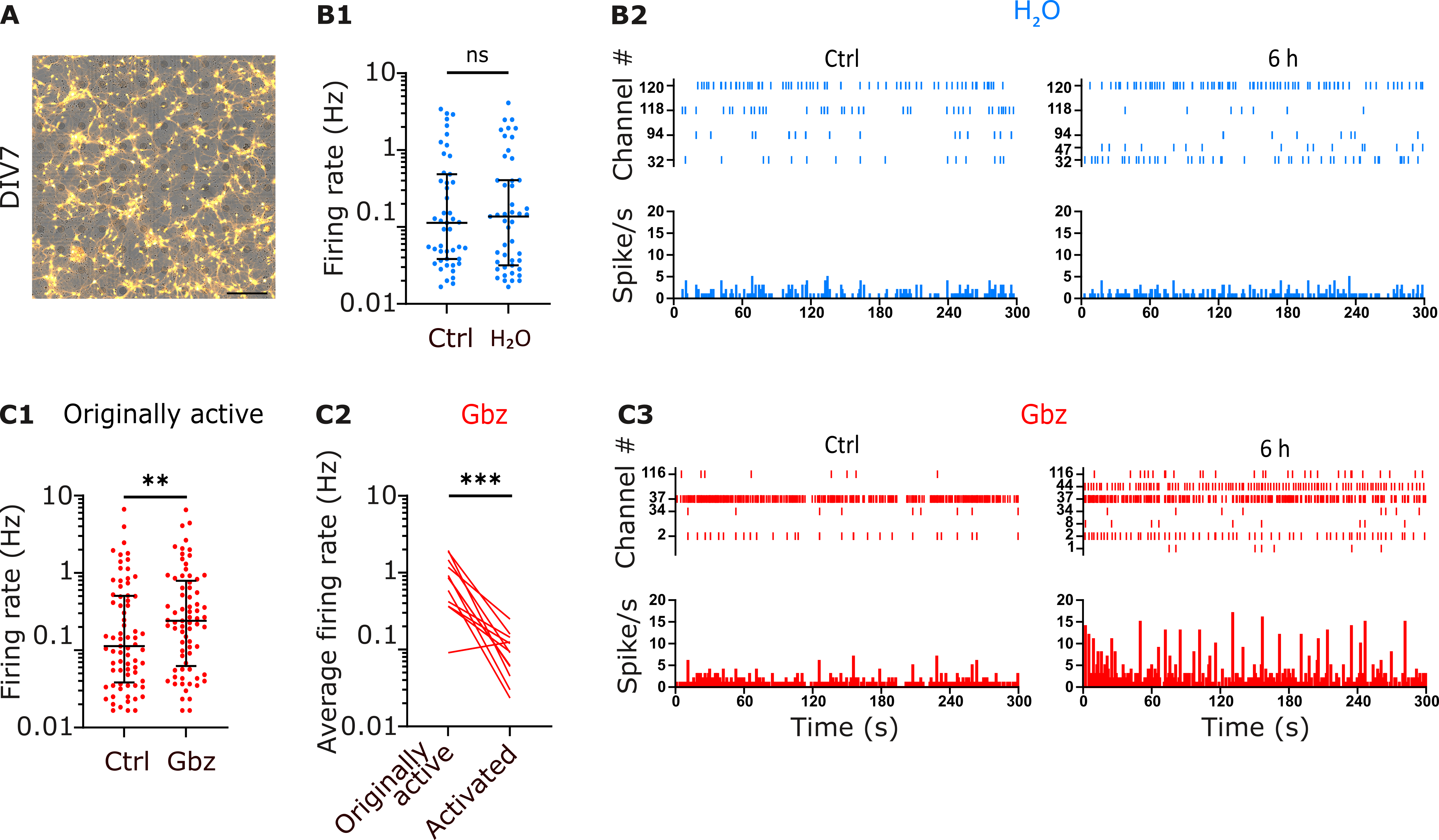

Supplement: Supplementary_Figure_2_bhad210 [file supplementary_figure_2_bhad210.zip › Supplementary_Figure_2_bhad210.tiff]

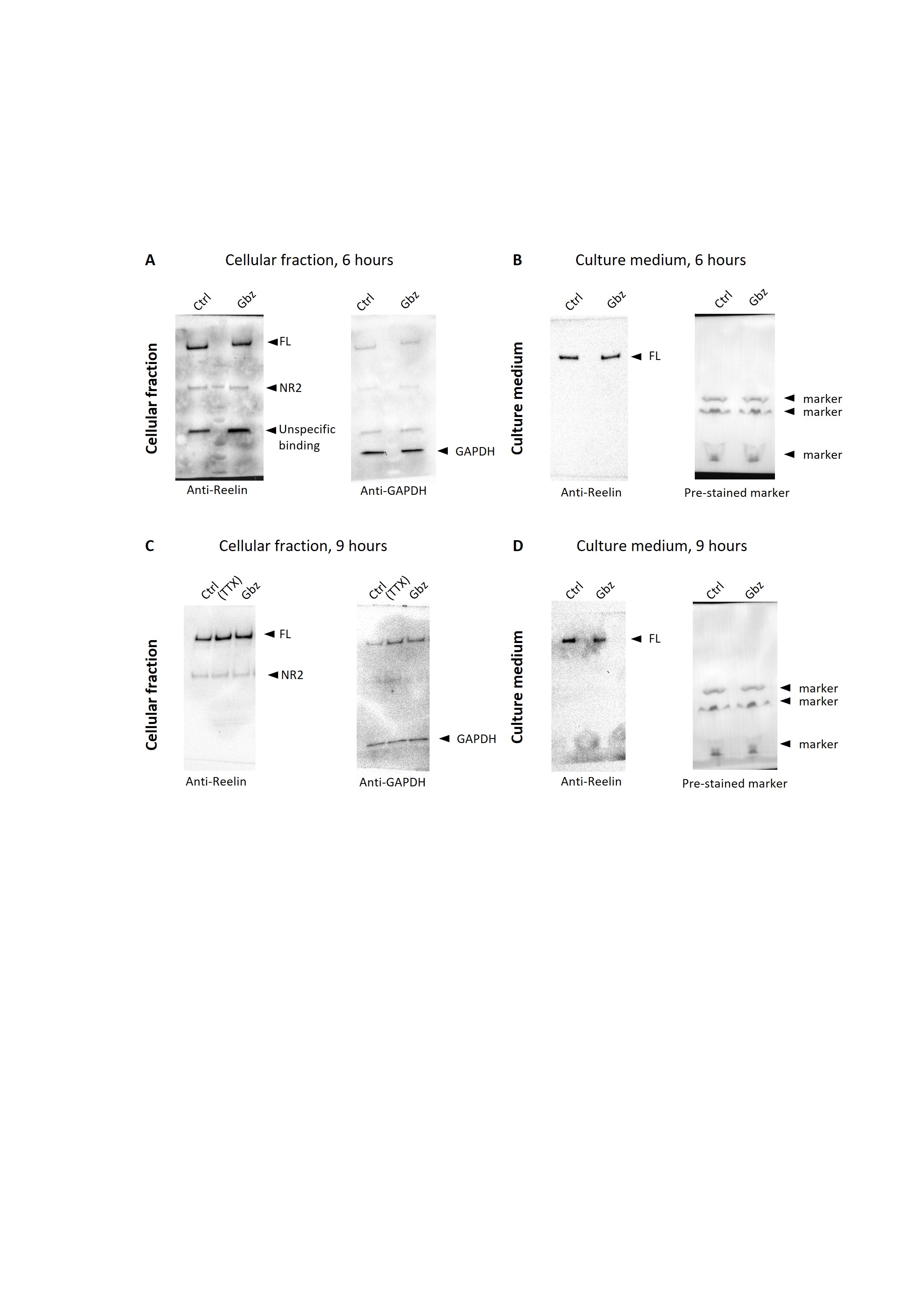

Supplement: Supplementary_Figure_4_bhad210 [file supplementary_figure_4_bhad210.jpeg]

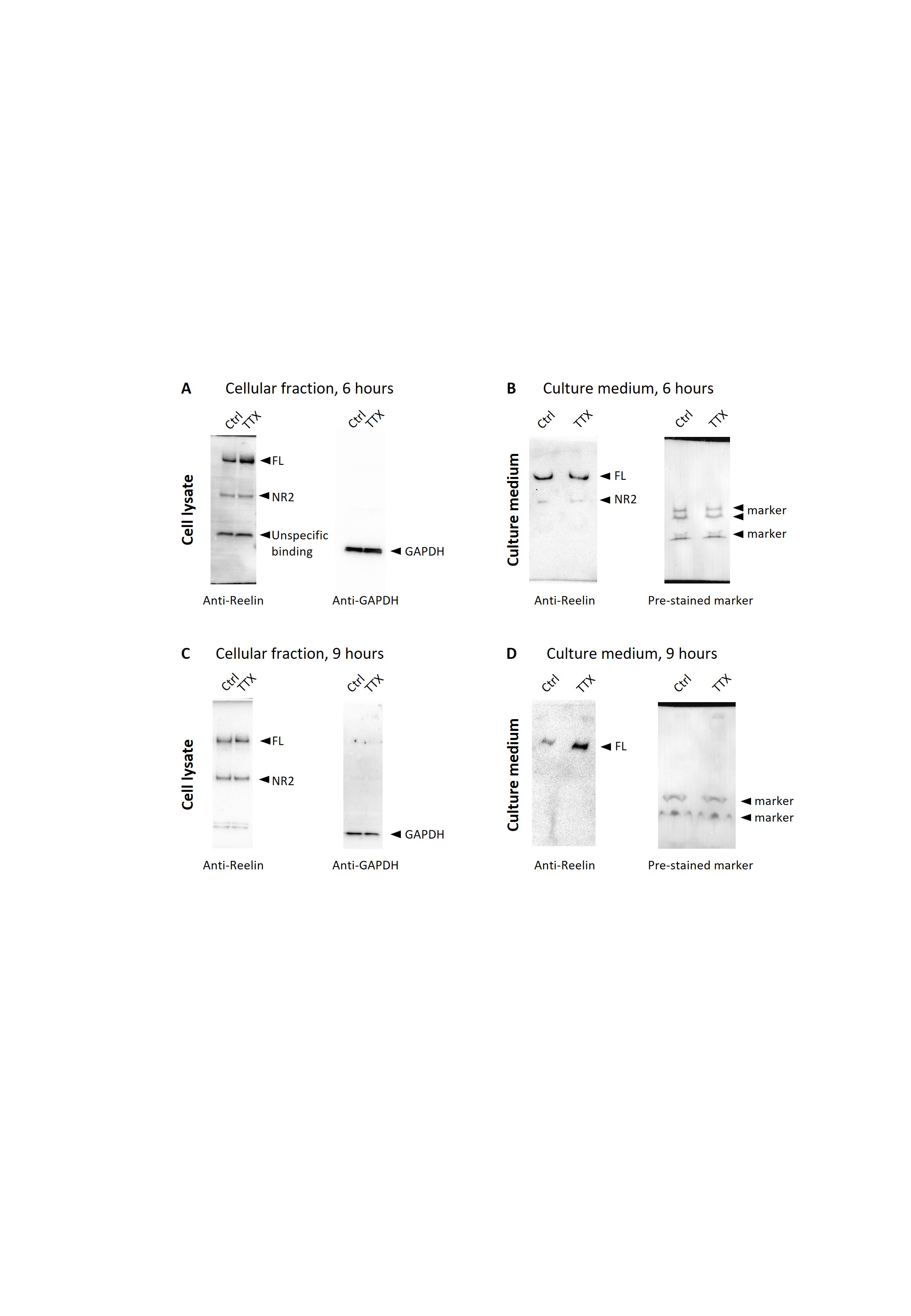

Supplement: Supplementary_Figure_6_bhad210 [file supplementary_figure_6_bhad210.jpeg]
